# Supplementary material for: Genome-Wide Association Analyses Identify Variants in IRF4 Associated With Acute Myeloid Leukemia and Myelodysplastic Syndrome Susceptibility
Source: Front Genet. 2021 Jun 17;12:554948. doi: 10.3389/fgene.2021.554948 (PMC8248805; doi:10.3389/fgene.2021.554948)
Supplement: Supplementary file 1 [file Data_Sheet_1.docx]

**Supplemental Tables and Figures**

**(Contains: Supplemental Table 1, Supplemental Figure 1, Supplemental Figure 2, Supplemental Figure 3, Supplemental Figure 4)**

*“Genome-wide association analyses identify variants in IRF4 associated with acute myeloid leukemia and myelodysplastic syndrome susceptibility”*

Junke Wang, Alyssa Clay-Gilmour, Ezgi Karaesmen, Abbas A Rizvi, Qianqian Zhu, Li Yan, Leah M. Preus, Song Liu, Yiwen Wang, Elizabeth Griffiths, Daniel O Stram, Loreall Pooler, Xin Sheng, Christopher A Haiman, David Van Den Berg, Amy Webb, Guy Brock, Stephen Spellman, Marcelo Pasquini, Philip McCarthy, James Allan, Friedrich Stölzel, Kenan Onel, Theresa Hahn and Lara Sucheston-Campbell*

Original Research, Front. Genet. - Applied Genetic Epidemiology

Received on: 23 Apr 2020, Edited by: Oskar A Haas

Manuscript ID: 554948

Keywords: GWAS, Leukemia, IRF4, Subset analysis, pleiotropy

**Supplemental Table 1.**

| **Supplemental Table 1. DISCOVeRY-BMT Acute myeloid leukemia (AML) / Myeloid dysplastic syndrome (MDS) Patient and Control Characteristics** | | |
| --- | --- | --- |
| Patient and Donor Characteristics | Cases Cohort 1 / Cohort 2  N= 1627 (%) / 682(%) | Controls Cohort 1 / Cohort 2  N= 2052 (%) / 762(%) |
| **Age, years** |  |  |
| Median (range) | 50 (<1-74.5) / 52 (<1-78) | 33 (18-61) / 31 (18-60) |
| **Sex** |  |  |
| Males | 886 (54) / 371 (54) | 1396 (68) / 553 (73) |
| Females | 741 (46) / 312 (46) | 656 (32) / 209 (27) |
| **Disease^1^** |  |  |
| ***AML, all cases*** | **1282 (79) / 487 (71)** | **-** |
| *de novo AML* | 1164 (72) / 454 (66) | - |
| *de novo AML with normal cytogenetics* | 373 (23) / 170 (25) | - |
| *de novo AML with abnormal cytogenetics* | 595 (37) / 241 (35) | - |
| *By Cytogenetic Subtype^2^:* |  |  |
| Core Binding Factor | 67 (11) / 32 (13) | - |
| MLL | 72 (12) / 48 (20) | - |
| Ph+ (t9;22) | 5 (1) / 1 (0) | - |
| APL (t15;17) | 18 (3) / 3 (1) | - |
| Any translocation | 97 (15) /35 (15) | - |
| Trisomy 8 | 103 (17) / 22 (9) | - |
| Trisomy 13 | 13 (2) / 3 (1) | - |
| Trisomy 21 | 23 (4) / 10 (4) | - |
| Trisomy 22 | 16 (3) / 11 (5) | - |
| Del5/del7 | 123 (21) / 55 (23) | - |
| Any Trisomy | 195 (33) / 92 (38) | - |
| Any Monosomy | 153 (26) / 50 (21) | - |
| >3 cytogenetic abnormalities | 213 (36) / 88 (37) | - |
| *Therapy-related AML* | 113 (7) / 33 (5) | - |
| *By Prior Diagnosis^2^:* |  |  |
| Breast Cancer | 39 (35) / 12 (36) | - |
| Non-Hodgkin Lymphoma | 20 (18) / 3 (9) | - |
| Hodgkin Lymphoma | 11 (10) / 3 (9) | - |
| Sarcoma | 9 (8) / 3 (9) | - |
| Gynecologic Cancer | 6 (5) / 2 (6) | - |
| Acute Lymphocytic Leukemia | 4 (4) / 2 (6) | - |
| Testicular Cancer | 4 (4) / 2 (6) | - |
| Other disease | 20 (18) / 4 (12) |  |
| ***MDS, all cases*** | **345 (21) / 195 (29)** | - |
| *By Sub disease^2^:* |  |  |
| MDS-unclassified^3^ | 58 (17) / 35 (18) | - |
| Refractory anemia | 77 (22) / 19 (10) | - |
| RAEB 1 and 2^4^ | 153 (44) / 89 (46) | - |
| Chronic myelomonocytic leukemia | 42 (12) / 16 (8) | - |
| RCMD, RCMD-RS | 0 (0) / 25 (13) | - |
| RARS | 14 (4) / 9 (5) | - |
| *de novo MDS* | 294 (18) / 150 (22) | - |
| *Therapy-related MDS* | 51 (3) / 45 (7) | - |
| *By Prior Diagnosis^2^:* |  |  |
| Non-Hodgkin Lymphoma | 15 (29) / 12 (27) | - |
| Breast Cancer | 8 (16) / 7 (16) | - |
| Acute Lymphocytic Leukemia | 4 (8) / 4 (9) | - |
| Hodgkin Lymphoma | 6 (12) / 2 (4) | - |
| Acute Myeloid Leukemia | 4 (8) / 4 (9) | - |
| Sarcoma | 1 (2) / 5 (11) | - |
| Chronic Lymphocytic Leukemia | 2 (4) / 3 (6) | - |
| Other disease | 10 (20) / 9 (20) | - |
| RAEB=Refractory Anemia Excess Blasts; RCMD=Refractory Cytopenia with Multilineage Dysplasia; RCMD-RS=Refractory Cytopenia with Multilineage Dysplasia and Ringed Sideroblasts; RARS=Refractory Anaemia with Ring Sideroblasts. | | |
| ^1^percentage of patients subgroup reflects the percentage of the total number of AML and MDS cases in each cohort; ^2^percentage of patients subgroup reflects the percentage of the cases of corresponding disease subgroups in each cohort; ^3^one individual had 5q-syndrome; ^4^6 individuals had RAEB in transmoration. | | |

| Supplemental Table 2. TWAS - Gene associations (P<4.0xE-04) | | | | | | | |
| --- | --- | --- | --- | --- | --- | --- | --- |
|  | Cohort 1 | | Cohort 2 | | Meta | |  |
| Gene | HR (95% CI) | P-value | HR (95% CI) | P-value | HR (95% CI) | P-value |  |
| *IRF4* | 3.6 (1.5,5.7) | 2.46E-05 | 4.9 (0.3,9.6) | 0.001 | 3.8 (2.4, 6.4) | **1.04E-07** |  |
| *AKT1* | 1.7 (1.2,2.1) | 2.51E-04 | 1.4 (0.8,1.9) | 0.139 | 1.6 (1.2,2.0) | **1.10E-04** |  |
| *NTN5* | 1.3 (1.1,1.4) | 0.001 | 1.3 (1.0,1.6) | 0.030 | 1.3 (1.2,1.4) | **1.14E-04** |  |
| *RASAL1* | 2.6 (0.8,4.4) | 0.006 | 4.0 (0.3,8.36) | 0.011 | 3.0 (1.7,5.3) | **2.29E-04** |  |
| *RCE1* | 1.5 (1.1,1.9) | 0.006 | 1.7 (1.0,2.5) | 0.012 | 1.6 (1.2,2.0) | **2.38E-04** |  |
| *RPH3A* | 1.4 (1.1,1.7) | 0.002 | 1.4 (0.9,1.9) | 0.044 | 1.4 (1.2,1.7) | **2.40E-04** |  |
| *YBEY* | 0.9 (0.8,0.9) | 0.001 | 0.9 (0.8,1.0) | 0.109 | 0.9 (0.8,0.9) | **3.55E-04** |  |
| *RASGRP2* | 3.7 (0.2,7.2) | 0.007 | 5.0 (2.1,12.2) | 0.026 | 4.05 (1.8,8.9) | **4.98E-04** |  |

**Supplemental Figure 1.** **Genome wide associations by cytogenetic subtype in DISCOVeRY-BMT**

Shown are the genome-wide P values by subtype from the meta-analysis of DISCOVeRY-BMT cohorts, including a total of 2158 AML and MDS cases and 2814 controls. The dashed horizontal line represents the suggestive threshold of P=5.0×10−6. The orange horizontal line represents the genome-wide significance threshold of P=5.0×10−8.

**
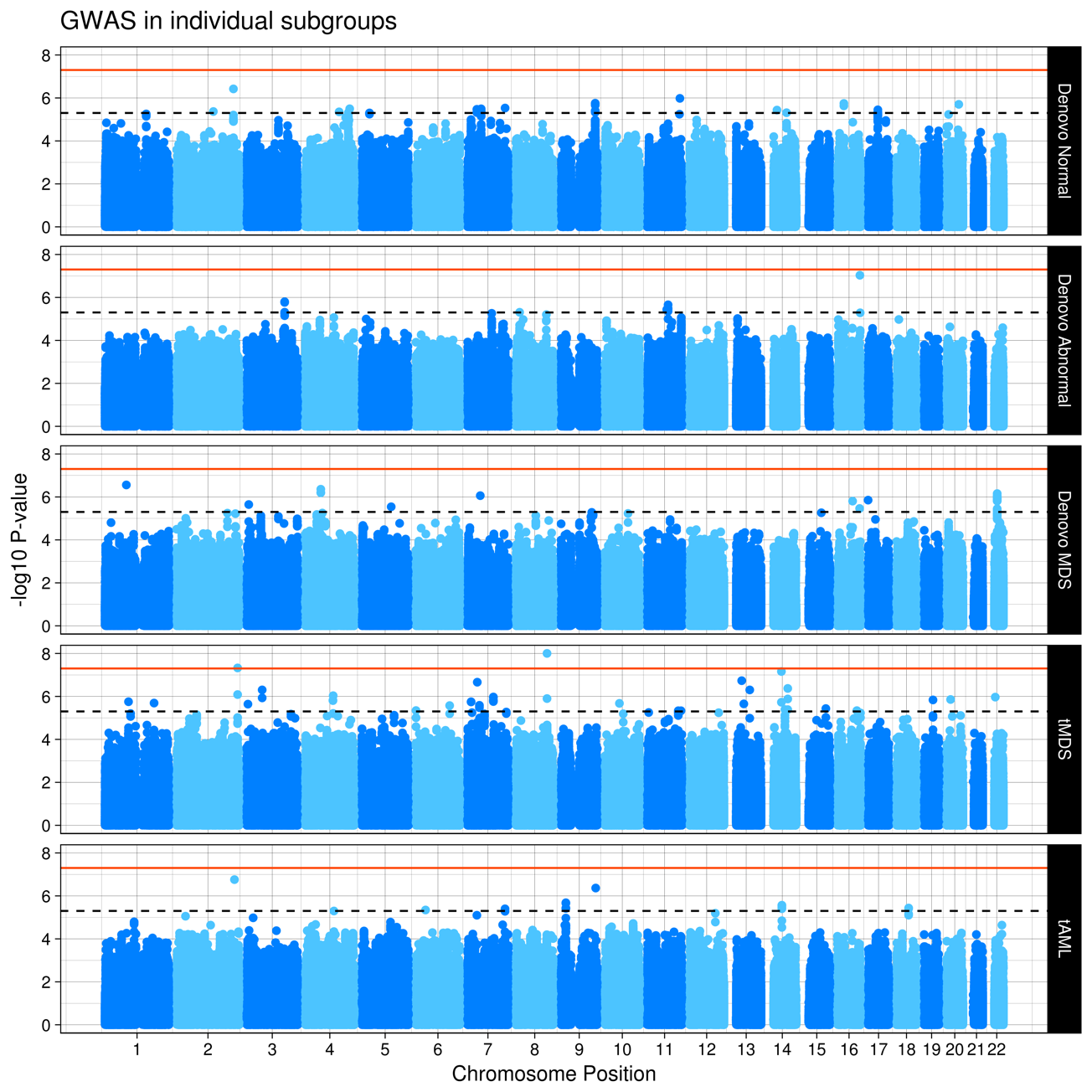
**

**Supplemental Figure 2. Significant chromatin interactions between the promoter region containing AML and MDS susceptibility variant, rs12203592 and the target region containing the previously identified CLL and HL susceptibility variant, rs9392017**

The circular plots show significant chromatin interactions between bait-target pairs, defined as a CHICAGO score >=5, designated with red arcs, generated by promoter capture HI-C experiments in multiple cell lines. Moving from the outside of the circles inward we see base pair position on chromosome 6 in Kb, protein coding genes are shown in grey (*HUS1B, EXOC2, DUSP22* and *IRF4*), the ENCODE roadmap epigenome chromatin states for (**LEFT**) E116: lymphoblastoid cell line and the following cell lines (**RIGHT**) E035:Primary hematopoietic stem cells; E036:Primary hematopoietic stem cells short term culture; E-50:Primary hematopoietic stem cells G-CSF mobilized Female; E-51:Primary hematopoietic stem cells G-CSF mobilized Male. This figure shows chromatin looping from the reference of the CLL and HL susceptibility region containing rs9392017 which illustrates this target region interacts with only few adjacent areas and only one transcriptional start site which contains rs12203592 providing support for the role of *IRF4* in CLL, HL, AML and MDS.


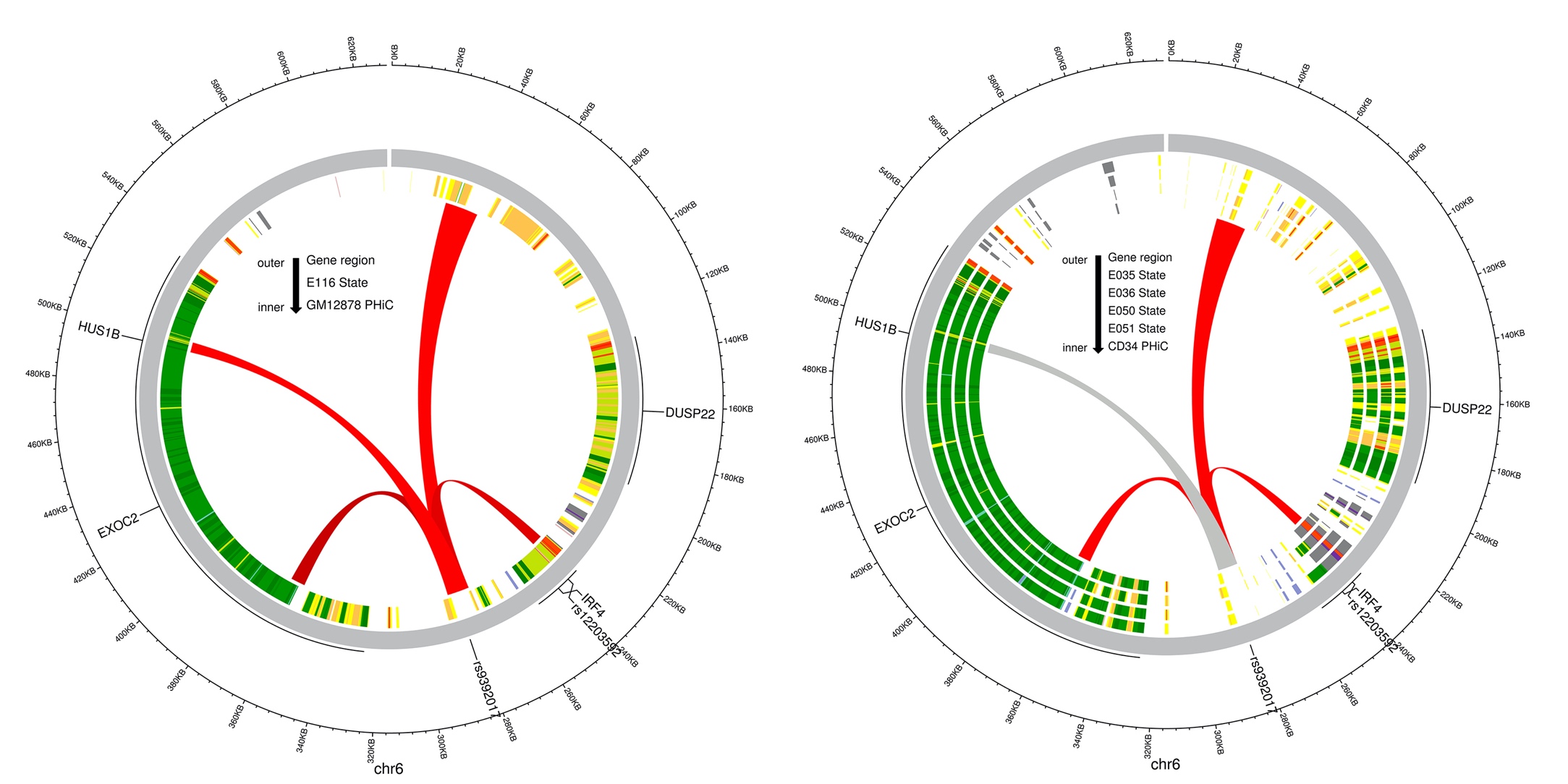


**Supplemental Figure 3. Genomic inflation factor for DISCOVeRY-BMT cohort 1 and 2 were 1.04 and 1.03, respectively. Quantile-quantile plots of SNPs after post-imputation quality control (MAF > 0.005, imputation quality scores > 0.8) showed very mild overdispersions of test statistics as compared to the null distribution.**

**
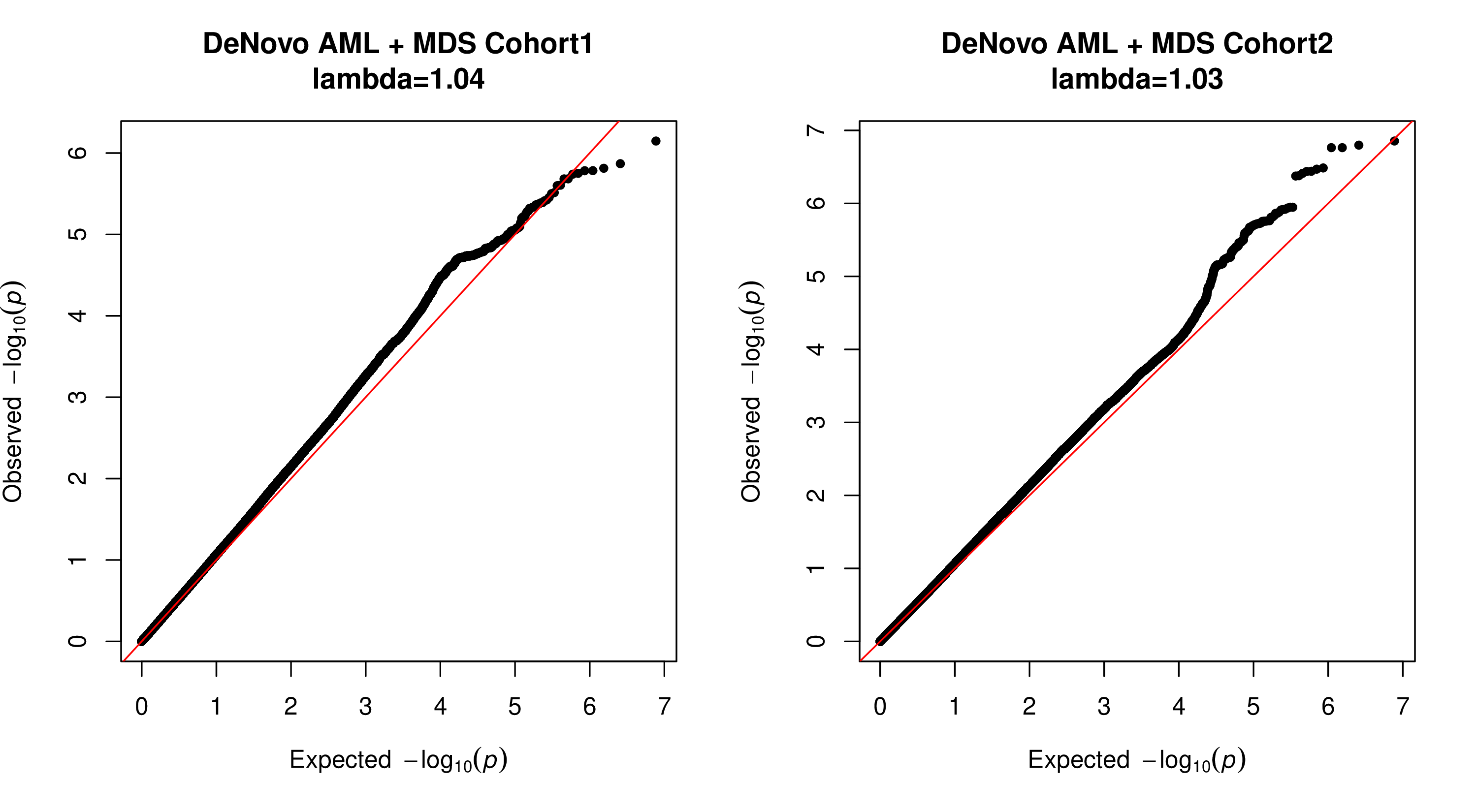
**

**Supplemental Figure 4. Expression of *IRF4* in TCGA AML samples compared to GTEx whole blood**


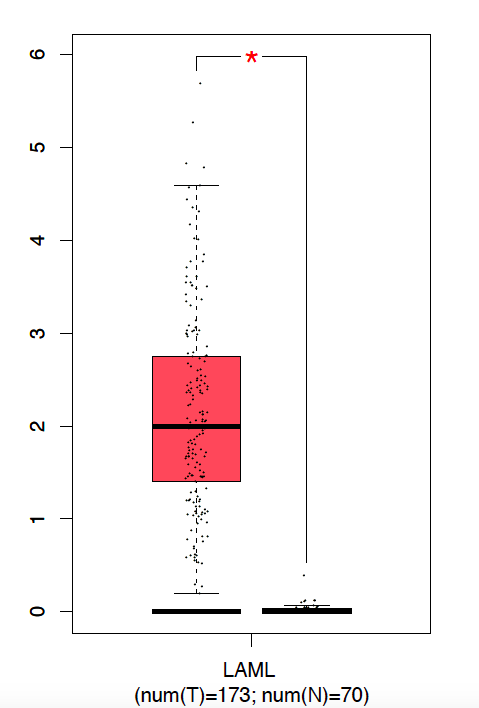


*IRF4* is expressed 1.75-fold more in AML samples

compared GTEx whole blood (P<.01)
